# Patient experiences with a prescription-based digital health application (DiGA) for knee disorders: a qualitative study in german orthopaedic care

**DOI:** 10.1186/s12891-026-10550-4

**Published:** 2026-10-28

**Authors:** Simone Burgert, Neven Raab, Tim Kornfeld, Johannes Knitza, Katharina Boy, Julian Schwarz, Susann May, Martin Heinze, Felix Mühlensiepen

**Affiliations:** 1 User Experience in Digital Health Lab, Center for Health Services Research, Faculty for Health Sciences, Brandenburg Medical School, 15562 Rüdersdorf, Germany; 2 Department of Orthopaedics and Trauma Surgery, Brandenburg Medical School, Immanuel Hospital Rüdersdorf, 15562 Rüdersdorf, Germany; 3 Institute for Digital Medicine, School of Medicine, Philipps-Universität Marburg, 35043 Marburg, Germany; 4 Center for Health Services Research, Faculty for Health Sciences, Brandenburg Medical School, 15562 Rüdersdorf, Germany; 5 Center for Mental Health, Brandenburg Medical School, Immanuel Hospital Rüdersdorf, 15562 Rüdersdorf, Germany; 6 Department of Cardiology, Angiology and Intensive Care Medicine, German Heart Center of the Charité, 13353 Berlin, Germany; 7 Univ. Grenoble Alpes, CNRS, Grenoble INP, LIG, Grenoble, 38000 SANGRIA France

**Keywords:** DiGA, Digital health applications, mHealth, Digital healthcare, Musculoskeletal disorders, Patients’ perspectives, Orthopaedics

## Abstract

**Background:**

Musculoskeletal disorders are among the leading causes of chronic pain, functional limitations, and reduced quality of life worldwide. Prescription-based digital health applications (DiGAs) have the potential to improve outpatient care, enhance treatment adherence, and address structural challenges such as shortages in physiotherapy services. However, real-world evidence on the use of DiGAs from the patient perspective in orthopaedic settings within the German healthcare context remains limited. This study aimed to explore acceptance, perceived potential, and challenges associated with DiGA use among patients with knee joint disorders.

**Methods:**

An exploratory qualitative study was conducted at an academic outpatient clinic for orthopaedics and trauma surgery in Germany. Ten adult patients (aged 18–65 years) with anterior cruciate ligament rupture or meniscal injury and a prescribed DiGA (“Orthopy”) were recruited. Semi-structured telephone interviews were conducted at the time of prescription (*n* = 10) and approximately two to three weeks later (*n* = 6; one follow-up interview took place after twelve weeks). All four participants who withdrew completed a dropout questionnaire. Data were analysed using qualitative content analysis according to Kuckartz. Main categories were derived deductively from the research question; subcategories were developed inductively from the data. Baseline and follow-up interviews of the six completing participants were compared within and across cases. Sociodemographic and clinical data were collected to contextualise findings. Trustworthiness was supported by iterative coding, an intra-coder consistency check, and team discussion of the coding frame. Coding was performed by a single researcher.

**Results:**

Participants were generally open to using the DiGA and valued its flexibility, location independence, and its role in bridging gaps in physiotherapy. The DiGA was primarily perceived as a complementary tool rather than a replacement for conventional therapy. Perceived key strengths included structured exercise guidance, educational content, and easy integration into daily routines, particularly in prehabilitation. Challenges included limited personalisation, insufficient adaptation of feedback, and limited motivational benefit when the app was used without accompanying professional guidance. Additional barriers comprised administrative delays in activation, usability issues among less digitally literate users, and insufficient physician guidance. Data privacy concerns played a minor role. Interviewed participants did not report serious safety concerns during the short observation period.

**Conclusions:**

Participants perceived the DiGA as a suitable complement to physiotherapy in orthopaedic care, particularly for prehabilitation and continuity of care. Better personalisation, usability, and integration into existing care structures could further enhance its perceived value. Long-term adherence, clinical effectiveness, safety, and sustainable implementation remain to be established. Future research should address these questions and patient-centred design.

**Trial registration:**

The study was registered in the German Clinical Trials Register (DRKS00036514) on 10 April 2025, after data collection had begun in January 2025 (retrospective registration).

**Supplementary Information:**

The online version contains supplementary material available at https://doi.org/10.1186/s12891-026-10550-4 .

## Introduction

Musculoskeletal diseases are among the leading causes of chronic pain, functional limitations, and reduced quality of life worldwide [1, 2]. In Germany, musculoskeletal diseases are likewise among the most frequent health problems and are associated with a considerable individual and economic burden [3–6]. Given demographic change and an ageing society, a further increase in disease burden is to be expected [1]. In particular, injuries to joint structures, such as those affecting the ligaments and menisci, represent a central risk factor for the development of degenerative secondary diseases such as knee osteoarthritis [7–11].

Digital health applications offer the potential to improve the care of patients with musculoskeletal conditions by making therapeutic services more accessible and by improving symptom management and adherence to therapeutic exercises [12–14]. This is particularly relevant in view of existing gaps in care provision – for example, a shortage of qualified physiotherapists and regional inequalities [15, 16]. Digital approaches include telemedical consultations, app-based training programmes, and digital rehabilitation services [12, 17]. At the same time, studies show that app-based rehabilitation approaches can achieve comparable or partly better results with regard to pain reduction and functional improvement than conventional forms of therapy [18, 19].

Despite proven effectiveness of digital interventions, the transfer into routine care remains a central challenge. The success of digital applications depends not only on their clinical effectiveness but substantially on their acceptance, integration into everyday care, and actual use by patients. Studies show that perceived usefulness, usability, as well as motivation are decisive for long-term use [4, 20, 21]. Thus, the patient perspective comes into focus as a central prerequisite for successful implementation: only applications that are accepted by users and integrated into their everyday life can realise their potential in routine care [22].

For digital health applications (DiGAs), which must demonstrate a positive healthcare effect within the framework of approval [23, 24], this aspect gains additional importance. Although the number of DiGAs in the field of musculoskeletal diseases is continuously increasing [25], as is the evidence regarding their effectiveness and use in routine care [14, 26, 27], a large part of previous research primarily focuses on clinical endpoints or technical aspects, while differentiated insights into usage experiences, acceptance processes, and perceived barriers in routine care are still limited.

Qualitative studies provide initial indications of relevant factors such as individualised content, feedback mechanisms, and presentation of progress, but often remain limited to development or pilot contexts [21, 26]. There is a lack of in-depth insights from real-world care situations that capture both initial expectations as well as actual usage experiences over time and thereby enable a more comprehensive understanding of implementation processes.

This study adds to the literature in four ways. First, it examines a DiGA prescribed and used under routine reimbursement conditions in German orthopaedic care, not in a development or pilot setting. Second, it covers the full prescription pathway, from physician counselling and code activation to everyday use. Third, it links expectations at the time of prescription with reported experiences approximately two to three weeks later. Fourth, it retains structured information from all participants lost to follow-up and thereby offers insights into early attrition.

The qualitative DiGA@Ortho study therefore aims to explore acceptance, challenges, and perceived potential of DiGA use from the perspective of patients with knee joint diseases. By combining data collection at two time points, we aim to obtain a differentiated picture of expectations and usage experiences and to derive concrete starting points for user-centred development and successful implementation in routine care.

## Methods

### Study design, recruitment and setting

We conducted an exploratory qualitative interview study with patients prescribed a DiGA for knee joint disorders, reported in accordance with the COREQ checklist. The study took place at the university outpatient clinic for orthopaedics and trauma surgery of the Brandenburg Medical School university hospital in Rüdersdorf, Germany. Qualitative data were collected between January and June 2025.

Participants were recruited face-to-face; sampling was consecutive, including all eligible patients during the recruitment period. To support recruitment, a “How to DiGA” handout was distributed to the medical department, outlining the general advantages of DiGA use, diagnostic criteria, and the steps of prescription (see supplementary material). Physicians verified eligibility during routine consultations before providing study information.

Sampling was consecutive rather than dimension-driven: physicians verified eligibility and informed patients during routine consultations, and all eligible patients who expressed interest were subsequently invited by e-mail to take part in the interviews. The number of patients screened and the number who declined participation were not systematically recorded.

We did not use data saturation as a formal stopping criterion. Recruitment ended with the closing of the recruitment period; in addition, “Orthopy” was the only DiGA available for prescription for the included diagnoses, which limited the eligible population. During the final interviews no substantially new aspects emerged, which supported the decision to stop recruitment; we report this as an observation rather than a formal saturation claim. Sample size was informed by the concept of information power [28]. The research question was narrow, all participants used the same DiGA in a single clinical setting, and the analysis was descriptive. Under these conditions, a small sample can carry sufficient information. The short interviews, the absence of field notes, and the loss of four participants at follow-up limit the depth and the longitudinal coverage of the data; we return to this in the Limitations.

Patients were eligible if they were aged 18–65 years, under orthopaedic treatment for meniscus damage or anterior cruciate ligament rupture, the diagnoses covered by the app, had received a DiGA prescription, were proficient in German, and provided written informed consent.

The DiGA under investigation, “Orthopy” (www.orthopy.de), was listed in the German DiGA directory at the time of the study and approved for anterior cruciate ligament rupture and/or meniscus damage. It comprises educational content, physiotherapeutic training plans for home exercise, guideline-based exercises, and a visual display of therapy progress as a motivational aid [29]. The app can be used preoperatively during the waiting period until surgery and postoperatively for early rehabilitation, thereby serving tertiary prevention in the context of arthroscopic procedures. Training intensity is evaluated after each exercise session based on patient-reported parameters such as perceived exertion, local heat sensation, and pain; combined with patient-reported outcome measures (PROMs), this enables individual monitoring of progress. Orthopy can be used as a supplement to conventional physiotherapy or temporarily as a stand-alone therapy to bridge waiting times and close gaps in care.

### Data collection

The semi-structured interview guide was developed specifically for this study. Prior to data collection, it was piloted in two test interviews with individuals similar to the target group; based on these, the wording of the questions was adjusted to improve comprehensibility.

The interviews combined open and closed questions and addressed the following main topics: acceptance, benefits and barriers, as well as experiences with the prescription process, usability and handling, functionality, potential for improvement, and sustainability. The fully translated interview guide can be found in the supplementary material. All interviews were conducted by telephone (audio only) by SB, a physiotherapist and medical student on the DiGA@Ortho team without prior experience in qualitative data collection, from her home. No notes were taken during the interviews. SBs training and professional experience in physiotherapy enabled her throughout the project – and particularly during the development of the interview guide – to assess the practical relevance of the exercises, the need for daily practice and the importance of maintaining motivation, and to ask appropriate follow-up questions during the interviews. This dual role was not mentioned during the interviews, and the interview guide was followed. The transcription was carried out with the aid of AI, using the NoScribe software [30]. NoScribe processes audio locally; no audio files were transferred to third-party servers. Transcription followed a semantic, content-focused convention. All transcripts were manually checked by SB against the audio recordings and corrected where necessary; filler words and dialectal expressions were smoothed.

Before data collection, SB received training in qualitative interviewing and analysis provided by FM and SM, complemented by university courses on qualitative research methods and the conduct of interviews; FM and SM supervised data collection and analysis. Participants took the calls in a private setting of their choice; no other persons were present during the interviews. There was no prior relationship between interviewer and participants. Transcripts were not returned to participants, and participants did not comment on the findings. A short questionnaire was used to systematically record data on participants who withdrew from the study; among other things, it examined the specific reasons for withdrawal, such as time constraints or personal circumstances (see supplementary material).

### Data analysis

We analysed the data using structuring qualitative content analysis according to Kuckartz [31]. The three main categories – acceptance, challenges, and potential – were derived deductively from the research question and the interview guide. Subcategories were developed and revised inductively during engagement with the data, following the principles of rule-based category formation according to Mayring [32]. The analysis was carried out using MAXQDA software (Verbi GmbH, Berlin, Version MAXQDA24) [33].

The coding unit was a complete statement on one topic; the context unit was the full interview. A segment could receive more than one code when a statement served several analytic functions, for example ease of use (acceptance) and access to therapy (potential). Contradictory statements were coded and retained, and we actively searched for negative cases during category revision. Anticipated associations noted in the codebook, for example between technical proficiency and acceptance, were treated as analytic memos, not as coding rules.

In a second analytic step, we compared baseline and follow-up data. Because activation of the prescription took up to two weeks, four participants had already begun using the DiGA at the time of their first interview; expectation-related and experience-related statements were therefore separated at the level of coded segments rather than whole interviews. We separated the 16 interviews by time point and linked both interviews of the six completing participants. For each of these six cases, we constructed a within-case matrix contrasting expectations at prescription (T0) with reported experiences approximately two to three weeks later (T1). We then compared cases with each other and classified expectations as confirmed, modified, contradicted, or newly emerged. Baseline interviews of the four participants who did not complete follow-up contributed to the cross-sectional themes and to the descriptive comparison of completers and non-completers, but not to the longitudinal comparison. Quotations in the Results are labelled by time point. For one participant (ID9_f_62), the second interview took place approximately twelve weeks after the operation rather than after approximately two to three weeks; this case is reported descriptively and marked in Table 1.

**Table 1:** Patient demographics

ID	Age	Sex	educational qualification		Surroundings	Disease	Follow-up / Reason for dropout
*ID1_f_29*	29	f		university degree	Small city *	Other spontaneous ruptures of one or more ligaments of the knee joint: anterior cruciate ligament	Yes
*ID2_f_28*	28	f		Secondary school leaving certificate	Small city *	Acute meniscus tear	Yes
*ID3_m_45*	45	m		Completed vocational training	Small city *	Acute meniscus tear	Yes
*ID4_f_58*	58	f		university degree	Rural area **	Meniscus damage caused by an old tear or an old injury	No/ Not sufficiently informed about the potential benefits
*ID5_f_36*	36	f		Completed vocational training	Small city *	Other spontaneous ruptures of one or more ligaments of the knee joint: anterior cruciate ligament	No/ Acute private emergency (water damage in the building)
*ID6_f_47*	47	f		Secondary school leaving certificate	Small city *	Other spontaneous ruptures of one or more ligaments of the knee joint: anterior cruciate ligament	No/ Sufficient physiotherapy
*ID7_m_55*	55	m		Completed vocational training	City with a population of over a million****	Meniscus damage caused by an old tear or an old injury	No/ Lack of time
*ID8_f_61*	61	f		Secondary school leaving certificate	medium-sized city***	Acute meniscus tear	Yes
*ID9_f_62*	62	f		university degree	Rural area **	Acute meniscus tear	Yes
*ID10_f_48*	48	f		Completed vocational training	Small city *	Acute meniscus tear	Yes

*Small city : 5,000–20,000 inhabitants; **Rural area: municipality with fewer than 5,000 inhabitants; ***Medium-sized town (20,000–100,000 inhabitants); ****City with over a million inhabitants (over 1,000,000 inhabitants)

To ensure the trustworthiness of the qualitative analysis, several measures were taken. All coding was performed by one researcher (SB). In the early phase of the analysis, SB recoded a subset of the initially coded interviews after an interval of approximately two weeks to check the stability of the coding frame and the fit of the interview guide; discrepancies led to refinements of category definitions. Because the coding frame remained substantially stable, this check was not repeated for later interviews, and the proportion of recoded material was not formally documented. We did not calculate an agreement coefficient, and no independent second coder was involved. The development and structuring of the category system were regularly discussed and reflected within the research team (FM, male, SB, female and NR, male). Changes to the coding tree were documented and transparently recorded. These discussions supported reflexivity; they do not constitute independent second coding. In addition, continuous reflection loops were conducted to critically examine the researcher’s role and potential influences on interpretation to increase the credibility and traceability of the results.

We selected representative quotes; SB translated them from German into English, and SB and FM jointly checked the translations for semantic equivalence. Paraphrased examples in the supplementary coding tree are marked as such; all quotations in the manuscript are direct translations. The manuscript was prepared in accordance with the Consolidated Criteria for Reporting Qualitative Research (COREQ) [34]; these, as well as the interview guide, coding tree, and coding guideline, can be found in the appendix.

### Supplementary quantitative measures

To contextualise the qualitative findings, supplementary questionnaires on quality of life and knee function were administered.

The EQ-5D-5 L (EuroQol 5 Dimensions 5 Level Version) is a generic instrument for measuring patient-reported outcomes (PROs). Its five subcategories—mobility, self-care, usual activities, pain/discomfort, and anxiety/depression—are each rated by patients on five levels (Level 1: no problems/pain/anxiety; Level 2: slight problems/pain/anxiety; Level 3: moderate problems/pain/anxiety; Level 4: severe problems/pain/anxiety; Level 5: extreme problems/pain/anxiety) [35].

Global health was additionally assessed with a single-item question (wording: “We want to find out how good or bad your health is today. To do this, we use the following scale, which is numbered from 0 to 10. Zero is the worst health you can imagine. 10 is the best health you can imagine. Please mark the point on the scale that best describes your health today”).

The International Knee Documentation Committee (IKDC) Subjective Knee Evaluation Form includes an assessment of the knee with regard to symptoms, sports activity, and knee function. Low percentage values indicate poorer function and more symptoms, whereas higher percentage values indicate better function and fewer symptoms [36].

All three instruments were administered at both interview time points (T0 and T1). Only the baseline values are reported (Table 2): the instruments serve to characterise the sample and its functional limitations at study entry, and with six completers a descriptive baseline–follow-up comparison would risk over-interpretation. We report the EQ-5D-5L as the five-digit descriptive health state; we did not calculate a country-specific index value because the data serve descriptive contextualisation only. IKDC raw scores were transformed to the 0–100 scale following the standard scoring instructions. All baseline questionnaires were complete, with no missing items. Given the sample size, we report descriptive results only and performed no inferential testing.

**Table 2:** Health-related characteristics and study participation

ID	EQ-5D-5 L Health State	Global Health	IKDC	+Physiotherapy (PT)
*ID1_f_29*	42,533	3/10	18%	+PT
*ID2_f_28*	31,131	7/10	56%	
*ID3_m_45*	32,331	6/10	16%	
*ID4_f_58*	31,441	6/10	25%	+PT
*ID5_f_36*	21,221	8/10	31%	
*ID6_f_47*	21,121	10/10	46%	
*ID7_m_55*	44,342	3/10	25%	
*ID8_f_61*	31,323	6/10	49%	
*ID9_f_62*	31,131	8/10	60%	
*ID10_f_48*	32,334	6/10	42%	+PT

Global Health Question: We want to find out how good or bad your health is TODAY. To do this, we are using the following scale, which is numbered from 0 to 10.;0 (zero) is the worst health you can imagine. 10 is the best health you can imagine.Please tick the point on the scale that best describes your health TODAY.;EQ-5D-5L Health State: Descriptive profile;+PT: plus physiotherapy

### Ethical considerations

The study was approved by the Ethics Committee of Brandenburg Medical School (241092024-BO-E) and registered in the German Clinical Trials Register (DRKS00036514) on 10 April 2025. Registration took place after data collection had begun in January 2025 and was therefore not fully prospective: the project was initially conceived as a small exploratory pilot; when it developed into a full study, a protocol was drawn up and the study was registered. No changes were made to the protocol after registration. All participants provided informed consent prior to participation. Participation was voluntary and data were processed in pseudonymised form.

### Results

### Participant characteristics

A total of 16 interviews were conducted with 10 participants, including ten interviews at the time of DiGA prescription and six follow-up interviews approximately two to three weeks later (one after twelve weeks). Participants who were interviewed both at the time of prescription and at follow-up are additionally marked in the baseline characteristics table. Four participants withdrew from the study after the first interview; all four completed a dropout questionnaire.

The interviews lasted on average 13 min and 35 s (min. 7 min 34 s / max. 19 min 46 s). All participants were prescribed the DiGA “Orthopy”, which was approved at the time of prescription [29].

Mean age was 47.1 years (SD 14.3; range 28–62). Eight out of ten participants (80%) were female. Six out of ten participants (60%) were from a small town, defined as 5,000–20,000 inhabitants. Three out of ten participants (30%) received physiotherapy in addition to the DiGA. The study population comprised five participants (50%) with an acute meniscal tear, two (20%) with meniscal damage secondary to a previous injury, and three (30%) with injuries affecting the knee ligament structures.

By integrating the EQ-5D-5 L, the Global Health and the IKDC into the table, we aim to provide the reader with further insight into the functional limitations caused by knee joint disease as well as a broader view of the participants’ health.

The interview guide as well as the coding tree were structured according to the three central aspects of acceptance, potential, and challenges and assigned to corresponding subcategories. The relevant documents can be found in the supplementary materials.

Four of ten participants (40%) withdrew from study follow-up after the first interview; all four completed the dropout questionnaire (see appendix). Withdrawal from study follow-up and discontinuation of DiGA use are distinct processes; the questionnaire covered both. Three of the four non-completers had begun using the DiGA and discontinued use as well as follow-up (one because concurrent physiotherapy was sufficient, one for lack of time, one in the context of an acute private emergency); one had not yet begun use because activation was still pending. In response to the question: “Why did you decide to withdraw from the DiGA study?”, one person cited an acute private emergency (water damage in the building), one person cited a lack of time, and one person felt they had not been sufficiently informed about the potential benefits of the study. One person was receiving adequate physiotherapy. Descriptively, completers (*n* = 6; five women, one man; aged 28–62 years; two with concurrent physiotherapy; baseline IKDC 16–60%) and non-completers (*n* = 4; three women, one man; aged 36–58 years; one with concurrent physiotherapy; baseline IKDC 25–46%) showed no discernible pattern of difference; a comparison including baseline expectations is provided in the longitudinal section below. We performed no inferential tests.

**Fig. 1:**
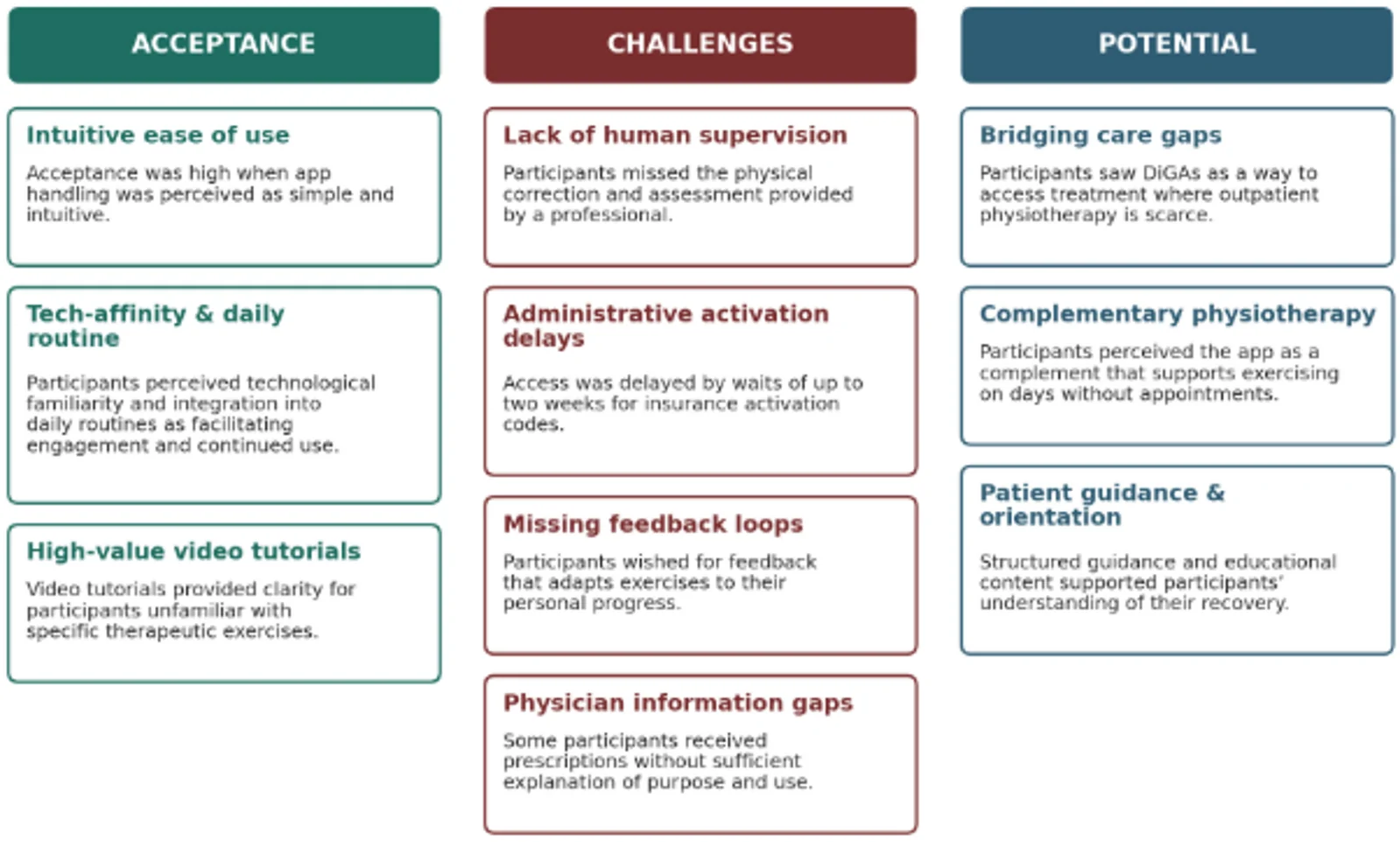
Conceptual framework: DiGA acceptance, challenges and potential

An overview of the main results is provided in Fig. 1.

The following presents the three main thematic blocks formulated in the research question: acceptance, challenges, and potential. The subordinate subcategories as well as the coding guideline can be found in the supplementary material.

#### Expectations of the DiGA and acceptance

Against the background of perceived bottlenecks in physiotherapeutic care, the interviews revealed a pronounced general openness towards digital health applications. However, this openness was less to be understood as technology-driven approval, but rather as a functional response to perceived deficits in the existing healthcare system. In this context, digitalisation was primarily framed to increase efficiency and to accelerate care processes, particularly with regard to the timely clarification of concerns.

“…to accelerate processes…*”* and to clarify matters more quickly than via analogue pathways….

*ID3_m_45*,* T0*,* pos. 20–21*.

At the same time, respondents referred to concrete structural problem situations, such as difficulties in obtaining appointments or limited telephone accessibility of practices, whereby digital applications were positioned not only as a supplement but implicitly also as compensation for existing care deficits.

The concrete acceptance of the DiGA was closely linked to its perceived usability and immediate practical benefit. The application was predominantly described as low-threshold and intuitively usable, which enabled uncomplicated use, especially after initial access steps such as receiving the activation code. A central factor for acceptance was the clear and structured presentation of content, particularly through video-based exercise instructions, which supported independent execution even without prior experience. The possibility of individual adaptation of exercises further strengthened the perceived fit to one’s own situation and thus facilitated integration of the application into everyday life.

“…that there is always a video so that you know which exercise it is… I find that very, very valuable.*”*

*ID1_f_29*,* T1*,* pos. 29*.

At the same time, potential access barriers were pointed out that could limit the reach of digital care services. Older patient groups were described as potentially disadvantaged, as a lack of digital competencies may make the use of the application more difficult and thereby increase the risk of selective exclusion from care services.

“…those who are now 80 years old in 2025… are not very familiar with digitalisation*”*

*ID2_f_28*,* T0*,* pos. 35*.

Digital health applications thus appear as a low-threshold supplement for a portion of patients, but at the same time may reinforce existing inequalities in access to care.

Within the field of acceptance, usage behaviour was also explored. In this context, a clear heterogeneity in usage behaviour among respondents became evident. Use varied both in terms of frequency and duration and ranged from an initial exploratory engagement to regular integration into everyday life. While some participants used the application routinely, for others it remained limited to more occasional or trial use.

These differences indicate that acceptance should not be understood as a static state but rather develops dynamically within the context of use and depends on individual preconditions as well as the perceived fit to one’s own situation.

Furthermore, a generally high level of technological affinity within the sample was noticeable, which can be interpreted as a central facilitating factor for the use and evaluation of the DiGA. Respondents predominantly described themselves as open towards technological innovations and particularly emphasised the gained temporal and spatial flexibility.

“…generally very open when it comes to technology… rather positive… where I don’t have to go somewhere.*”*

*ID1_f_29*,* T0*,* pos. 46*.

At the same time, this homogeneous technological affinity points to a potential bias in the results, as the reported high acceptance is likely not directly transferable to less technologically affine patient groups.

Overall, the results illustrate that acceptance of the DiGA is situated within a field of tension between structural care needs, individual usage prerequisites, and potential access barriers.

### Challenges

Challenges in dealing with the DiGA were particularly evident at the interfaces between medical care, administrative processes, and the actual use of the application. The prescription process itself was predominantly described as uncomplicated from both the medical and patient perspectives and could generally be implemented well with the provided information material. At the same time, individual reports indicated deficits in medical information provision: in particular, a lack of verbal communication regarding the functionality and purpose of the application was critically highlighted.

“…no information at all was given to me about what it is actually about….*”*

*ID5_f_36*,* single interview*,* pos. 13*.

This indicates that formal prescription alone does not necessarily go along with a sufficient understanding of the application and that its integration into the care context largely depends on the quality of medical communication.

Challenges were also identified at the administrative level that may delay access to the application. In particular, the activation of the access code by health insurance providers was perceived by many respondents as delayed in time and often extended over a period of up to two weeks.

“…ten working days….*”*

*ID5_f_36*,* single interview*,* pos. 8–9*.

Such delays contrast with expectations of fast and low-threshold digital care and may hinder immediate initiation of use.

In the context of use itself, further challenges emerged regarding the integration of the application into existing routines. Some participants reported performing the exercises independently of the app, especially when these were already familiar, while documentation within the application was perceived as an additional effort. Moreover, a lack of responsiveness of the application was criticised, particularly about individual feedback: the expectation that entered feedback would lead to an adaptation of content was partly not fulfilled.

“…if I indicate that the exercises are too easy…, then I would expect some alternative exercises to be suggested….*”*

*ID5_f_36*,* single interview*,* pos. 46*.

This points to a discrepancy between expectations of adaptive, personalised applications and their actual functionality.

Issues of data security and integration into overarching digital infrastructures, such as the electronic patient record, were predominantly assessed pragmatically by respondents. While a majority of participants generally agreed to the use of their data, a certain uncertainty regarding the specific use and control over their own data was also evident.

“…my data may be used… but I find it difficult to imagine that anything negative would be done with it….*”*

*ID2_f_28*,* T0*,* pos. 45*.

This indicates an ambivalent relationship between trust and lack of transparency.

Interviewed participants did not report serious safety concerns during the short observation period. The interviews did not include a systematic adverse-event assessment. Occasional minor complaints were classified as expected side effects and did not lead to discontinuation of use. At the same time, it became clear that the safe execution of exercises largely relies on the users’ own responsibility, particularly regarding the individual assessment of physical limits.

“…no one goes beyond their normal limit….*”*

*ID3_m_45*,* T1*,* pos. 34–35*.

### Potential

The results demonstrate a diverse potential of the DiGA, particularly with regard to a temporally and spatially flexible extension of physiotherapeutic care. A central aspect was the possibility to use the application independently of fixed appointments and locations, thereby facilitating low-threshold integration into everyday life. This flexibility was described as particularly relevant in the context of limited mobility or restricted availability of physiotherapy services. At the same time, the use of the DiGA prior to surgical interventions opens the perspective of digital prehabilitation, thereby enabling patients to be involved in their treatment process at an early stage.

In interaction with conventional physiotherapy, the DiGA was predominantly perceived as a complementary instrument. In particular, the possibility to bridge therapy-free periods and to continue exercises independently was highlighted as an advantage, as this allows continuous exercise practice even outside physiotherapy sessions.

“But through the app, on the days when I can’t go, I can still get my knee moving. So I don’t have to give up any exercise.*”*

*ID6_f_47*,* single interview*,* pos. 46*.

The application was thus understood less as a replacement and more as a supplement to existing care.

“I would see it as a good combination, linking the app and physiotherapy equally.*”*

*ID6_f_47*,* single interview*,* pos. 46*.

At the same time, it became clear that this form of independent use reaches its limits, particularly in the case of individual problems or lack of external supervision, making accompanying professional guidance necessary.

A further potential lay in overcoming structural access barriers to physiotherapy. Eliminated travel distances and the possibility of location-independent exercise execution were perceived as relieving, particularly for patients with limited mobility or insufficient access to care services.

“…there are various situations in which getting to physiotherapy is difficult or not possible.*”*

*ID2_f_28*,* T0*,* pos. 52*.

Digital applications can thus contribute to improving continuity of care, particularly in situations where conventional services are only limitedly available.

With regard to the content design of the DiGA, the high proportion of educational content was particularly highlighted positively, even though this did not initially correspond to users’ expectations. While expectations were primarily focused on concrete exercise instructions, the additional provision of knowledge led to a better understanding of one’s own rehabilitation process.

“…a better understanding of what was being done….*”*

*ID3_m_45*,* T1*,* pos. 15*.

The structured and professionally prepared presentation of content was perceived as an added value compared to freely available information sources.

“Of course, you can also look up exercises on YouTube, but this is much more condensed and better explained professionally.*”*

*ID3_m_45*,* T1*,* pos. 38–41*.

Some participants anticipated using the DiGA beyond the acute rehabilitation phase. They described it not only as short-term support in the recovery process, but also as a possible instrument for maintaining and improving physical function. Whether such use occurred was not observed in this study.

“It is not only interesting in terms of recovery, but also with regard to long-term improvement and strengthening of the knee.*”*

*ID3_m_45*,* T1*,* pos. 38–41*.

In the participants’ view, digital applications may contribute to independent health management beyond immediate therapy.

At the same time, an ambivalent picture emerged regarding motivation and use. While low-threshold availability and ease of implementation had a motivating effect for some participants, others described the lack of external control as a hindrance. The DiGA could thus function both as a structuring element in everyday life and reach its limits when intrinsic motivation or external guidance was lacking.

Use of the DiGA was only partially described by participants as intrinsically motivating. Rather, the application was perceived as a supportive instrument that primarily contributes through external structuring and guidance for performing exercises. Independent motivation enhancement through the app itself was reported only to a limited extent.

“For me, the DiGA is something I can hold on to. I always need someone who gives me some orientation and tells me which exercises to do because they are good for me physically.*”*

*ID8_f_61*,* T1*,* pos. 32–33*.

This underlines the importance of individual prerequisites for the successful use of digital applications.

Regarding the actual use of content, it was shown that the independent compilation of training plans was generally perceived as intuitively feasible but could be associated with uncertainty regarding the selection of appropriate exercises. Responses differed clearly depending on whether prior knowledge and previous experience with knee joint diseases were present.

“I put together the training by looking at the exercises available and selecting what suited me. I chose the exercises freely. I have some prior knowledge from the fitness sector and discussed it with my physiotherapist.*”*

*ID1_f_29*,* T1*,* pos. 14–17*.

While elements of training control, such as querying pain or swelling, were evaluated positively, the perceived individualisation of content remained partly limited.

In the participants’ view, the potential of the DiGA lies in flexible, complementary support of the rehabilitation process. Participants saw its usefulness as depending on individual usage competence, motivation, and integration into existing care structures.

### From expectations to experiences: within- and across-case comparison

For the six participants interviewed at both time points, we compared expectations at prescription (T0) with reported experiences approximately two to three weeks later (T1) and classified each expectation as confirmed, modified, contradicted, or newly emerged (Table 1). For one of the six (ID9_f_62), the second interview took place around twelve weeks after surgery; this case contributes descriptively. Owing to delays in code activation, the length of actual use at T1 varied considerably between cases, from several weeks of regular use to a few days of initial exploration.

#### Confirmed expectations

The expectation that the DiGA would serve as a complement to, not a replacement for, physiotherapy was expressed by all six participants at T0 and was not revised by any of them at T1. Where physiotherapy was available, hybrid use was enacted rather than merely endorsed:

*“I put together the training by looking at the exercises available and selecting what suited me. I chose the exercises freely. I have some prior knowledge from the fitness sector and discussed it with my physiotherapist.” ID1_f_29*,* T1*.

Expectations of structured, video-based exercise guidance were likewise confirmed; participants described the exercise videos and the guided selection as the core value of the application. The flexibility expected at T0 — exercising independently of appointments and locations — materialised for those who reached regular use, and one participant summarised the overall experience at T1 despite persisting criticisms:

*“But apart from that*,* it is definitely something that helps me.” ID8_f_61*,* T1*.

#### Modified expectations

Two expectations changed shape rather than being confirmed or refuted. First, the educational content exceeded expectations: participants had anticipated exercise instructions, not knowledge content, and described the latter as an unexpected gain in understanding at T1 (“a better understanding of what was being done”, ID3_m_45, T1; “It also makes you a bit curious.“, ID3_m_45, T1). Second, motivation turned out to be slower and more conditional than anticipated: one participant reported feeling motivated by the application for the first time only at the end of the follow-up period, and explicitly missed any reaction from the application when sessions were skipped. In addition, participants without concurrent physiotherapy (ID3, ID8) found themselves using the DiGA as their only structured support — a stand-alone role they had not sought, which sharpened rather than removed their wish for professional guidance.

#### Contradicted expectations

Two expectations were consistently contradicted. First, the expectation of fast, low-threshold digital access was contradicted in every case in which activation was discussed: participants waited around ten working days to two weeks for their activation code (“ten working days”, ID5_f_36, single interview; “No — I think I waited two weeks.“, ID2_f_28, T1). For ID2, the waiting time compressed the use window to such an extent that at T1 the application had only just been explored and the planned preoperative use had effectively been prevented; this case demonstrates that the barrier operates at system level, before any property of the application itself becomes relevant. Second, the expectation that entered feedback would have consequences — adaptive content, alternative exercises, a reaction to skipped sessions — remained unmet (see the statement of ID5_f_36 quoted above under Challenges).

#### Newly emerged themes

Three themes appeared at T1 without a counterpart at T0: [1] documentation burden and plan rigidity — confirming each repetition individually, being unable to adjust the number of sets, or losing exercises from the plan without a way to restore them (ID1, ID8, ID10); [2] technical faults such as crashes and interrupted exercise videos (ID10); and [3] self-managed progression as a safety grey zone: without professional supervision, participants advanced between exercise phases by trial and error (ID10) or compensated by calling the manufacturer’s support line (ID8). The application’s post-exercise queries on pain and swelling were valued, but did not replace the reassurance of professional feedback. 

#### Non-completers

The four participants who did not complete follow-up contributed baseline and, in three cases, early-use data. Of the four, one discontinued because concurrent physiotherapy was sufficient, one cited lack of time, one experienced an acute private emergency (water damage), and one — who had not yet obtained access at the time of the interview — felt insufficiently informed and framed withdrawal as a private decision. Three of the four had begun using the DiGA; two of them nevertheless rated it as helpful in the dropout questionnaire and stated they would use it again. Descriptively, non-completers did not differ from completers in technological affinity or in their evaluation of the application; their withdrawal was driven predominantly by external circumstances rather than by rejection of the DiGA. Attrition therefore does not appear to have selected for participants with more favourable attitudes towards the application, although the further course of the non-completers’ use remains unobserved. A detailed within- and across-case comparison of participants’ expectations and reported experiences is provided in Table 3.

**Table 3:** Within- and across-case classification of expectations (T0) against reported experiences (T1)

Expectation domain (T0)	ID1	ID2	ID3	ID8	ID9*	ID10
Complement to physiotherapy, not replacement	C	C†	M	M	C	M
Structured, video-based exercise guidance	C	n.t.	C	C/M	C	C
Educational content (anticipated at T0 by none)	–	–	E	–	–	–
Fast, low-threshold access	X	X	X	X	X	X
Adaptive feedback / personalisation	X	n.t.	n.s.	X	n.s.	X/M
Motivation through the application	M	n.t.	M	M	C	M
Safety and self-management confidence	C	n.t.	C	X	n.s.	M
Newly emerged at T1: documentation burden / plan rigidity	+			+		+
Newly emerged at T1: technical faults						+
Newly emerged at T1: self-managed progression / support workarounds				+		+
Newly emerged at T1: intention to use beyond rehabilitation			+			

C = confirmed; M = modified; X = contradicted/unmet; E = exceeded expectations; + = newly emerged at T1; n.t. = not testable at T1 (activation delay compressed the use window); n.s. = not salient in this case; † attitude maintained, use itself not yet testable. * ID9: second interview approximately twelve weeks after surgery; classification based on the first interview (early use) and reported descriptively

## Discussion

The aim of this study was to explore patients’ perspectives on the acceptance, challenges, and perceived potential of using a digital health application (DiGA) for knee joint disorders. The results show that the challenges lie less in the fundamental acceptance of the DiGA and more in practical implementation along care processes, user expectations, and structural conditions.

Compared with existing qualitative work on digital exercise interventions, this study contributes real-world evidence from the German DiGA prescription pathway in orthopaedic care. It links expectations at prescription with early usage experiences, documents barriers along the reimbursement and activation process, and preserves structured data from all participants lost to follow-up. Prior studies have largely examined development or pilot contexts [21, 26] or other indications [37].

The high level of technological affinity within the sample, along with the simultaneously identified potential access barriers for older patient groups, reflects known challenges in digital healthcare: The literature repeatedly emphasises that digital health literacy is a key predictor for the successful use and adherence to digital applications [38, 39]. Real-world studies from German rheumatology found that only 13% [40] and 15% [14] of patients, respectively, completed the full DiGA programme. Consequently, there is a risk that DiGAs may not reduce existing inequalities in healthcare, but could potentially even exacerbate them if no targeted support measures are implemented for groups with lower digital literacy [41].

Regarding the integration into healthcare provision, the results underline the importance of hybrid care models. The DiGA was predominantly perceived as a supplement to physiotherapeutic treatment, which is in line with previous studies describing the benefits of digital applications particularly in combination with conventional therapy [42]. At the same time, this combination addresses a central structural problem in healthcare, namely the limited availability of physiotherapeutic services as well as associated waiting times and mobility barriers [43]. The literature suggests that digital interventions have particular potential where care bottlenecks exist and continuous support would otherwise be limited [44].

Another key finding concerns the limited perceived adaptability of the application. The desire for more personalised content and feedback mechanisms suggests that current digital applications often do not sufficiently respond to individual progress. The literature describes personalisation and adaptive feedback systems as central areas of development for improving adherence and clinical effectiveness of digital interventions [44] and this has been previously criticised by patients using back pain DiGAs [37].

The repeatedly expressed preference for additional human support highlights that digital applications should not be considered in isolation. Rather, the results confirm the need for hybrid care in which digital and personal support interact complementarily. Previous work also shows that, particularly in musculoskeletal conditions, the combination of digital guidance and therapeutic support can improve acceptance and use of digital applications [42, 45].

In the broader context of digital healthcare, the integration of DiGAs into routine care remains a key challenge. While digital therapeutics have the potential to reduce the burden on labour-intensive healthcare services and strengthen patient participation through greater self-management, this “democratisation” of healthcare is accompanied by important challenges. From a healthcare science perspective, increased autonomy may also shift responsibility to patients, potentially exacerbating inequalities when digital health literacy and supportive care structures are lacking [27].

The results are also in line with existing evidence on the effectiveness of digital physiotherapeutic interventions. A recent meta-analysis shows significant but overall small effects of digital physiotherapy on physical function in knee osteoarthritis, with high heterogeneity across studies [46]. This underlines the need for further differentiated research on the specific effectiveness of individual digital applications and their implementation conditions [47].

The ‘Orthopy’ medical health app was removed from the BfArM’s DiGA register during the course of this study. The diagnoses covered by the ‘Orthopy’ app are not currently covered by any other DiGA, but occurred predominantly during in-person consultations as part of the study. This situation highlights a key methodological challenge in DiGA research, namely ensuring research continuity. In particular, the dynamic nature and vulnerability of the DiGA market make it difficult to conduct longitudinal studies on the use of DiGAs that have only been provisionally included in the DiGA register. Despite these circumstances, the reported results remain relevant. Because the specific product is no longer available, the value of the findings lies in transferable process issues across prescription-based digital rehabilitation: prescribing and counselling, code activation, patient education, professional support, digital health literacy, and integration with physiotherapy.

Finally, medical-ethical considerations indicate that digital applications should not be used as a substitute, but exclusively as a complement to guideline-based treatment. In addition, the importance of enhanced medical counselling and consideration of digital health literacy is emphasised. Concepts such as the “application anamnesis” can help to better integrate the use of digital therapeutics into clinical practice and enable patients to use them safely and independently [45].

### Limitations

The small sample size, the predominantly female sample, and the single-centre setting limit the transferability of the results. The group’s level of technical proficiency was high. The results should therefore be interpreted with this in mind, and future studies should focus on a targeted sample of individuals with lower levels of technical proficiency. As none of the dropouts cited the DiGA itself as the reason for dropping out – instead, other external factors played a role – it remains unclear how their user experience would have developed over time. Recruitment took place exclusively at one university centre. Patient profiles not captured in the sample – for example, patients with low digital affinity – further limit transferability. Screening and refusal numbers were not systematically recorded. The 40% loss to follow-up restricts the longitudinal comparison to six cases and may bias the follow-up findings towards participants with more favourable conditions for use. The qualitative analysis serves primarily to generate hypotheses and should not be interpreted as a definitive assessment of DiGA usage. The interviews were short (mean 13 min 35 s), and no field notes were taken; this limits the depth of the data. SB’s background in physiotherapy shaped the interview guide and the follow-up questions. The baseline guide asked directly whether the app could complement or replace physiotherapy; this framing may have prompted participants to describe the DiGA in relation to physiotherapy. Despite these limitations, the study provides rare insights into the implementation of DiGAs in routine practice.

All coding was performed by a single researcher. The intra-coder consistency check and the team discussions supported consistency, but they are not equivalent to independent second coding. This is a limitation of the analysis.

### Implications for practice and research

The results suggest that digital health applications (DiGAs) can make an important contribution as a complementary care measure for musculoskeletal disorders, particularly in the areas of prehabilitation and the provision of educational content. It is crucial that orthopaedic doctors provide guidance on the use of a DiGA and highlight both the possibilities and limitations for maintaining motivation and usage in the long term.

Furthermore, integration into existing healthcare systems, particularly into physiotherapy workflows, is crucial for increasing efficiency and acceptance. Physiotherapy professionals should be familiar with the relevant content of the DiGA, regularly review usage patterns for prescribed DiGAs, and provide accessible, personalised support to clarify any open questions and guide movement sequences. When integrated into home exercise programmes in physiotherapy, DiGAs are a time-efficient and low-threshold addition.

## Conclusion

This study suggests that patients with knee joint disorders are open to digital care approaches such as DiGAs and perceived the studied DiGA as a suitable complement to physiotherapy. Long-term adherence, clinical effectiveness, safety, and sustainable implementation could not be assessed within this design and remain subjects for future research. Future research should investigate systemic integration, long-term effects, interoperability with other digital solutions, and clinical outcomes. Attention should be paid to patients with lower digital health literacy and to the role of medical counselling in supporting sustained DiGA use.

## Supplementary Information

Below is the link to the electronic supplementary material.

Supplementary Material 1

Supplementary Material 2

Supplementary Material 3

Supplementary Material 4

Supplementary Material 5

Supplementary Material 6

## Data Availability

Data are available upon reasonable request. For further questions regarding the reuse of data, please contact the corresponding author (simone.burgert@mhb-fontane.de).

## Declarations

### Patient and public involvement

Patients and/or the public were not involved in the design, or conduct, or reporting, or dissemination plans of this research.
