# Supplementary material for: Patient experiences with a prescription-based digital health application (DiGA) for knee disorders: a qualitative study in german orthopaedic care
Source: BMC Musculoskelet Disord. 2026 Oct 28;27:953. doi: 10.1186/s12891-026-10550-4 (PMC13733015; doi:10.1186/s12891-026-10550-4)
Supplement: Supplementary file 1 [file 12891_2026_10550_MOESM5_ESM.pdf]

COREQ (COnsolidated criteria for REporting Qualitative research) Checklist

Manuscript: Patient Experiences with a Prescription-Based Digital Health Application (DiGA) for Knee Disorders: A Qualitative Study in German Orthopaedic Care (Ref: 2026-6232). Revised version. Locations are given as section references of the revised manuscript; items not carried out are marked accordingly.

Topic	Item No.	Guide Questions/Description	Reported in
**Domain 1: Research team and reflexivity**			
Interviewer/facilitator	1	Which author/s conducted the interview or focus group?	SB; Methods – Data Collection
Credentials	2	What were the researcher's credentials?	Title page; Methods – Data Collection
Occupation	3	What was their occupation at the time of the study?	Methods – Data Collection (physiotherapist and medical student)
Gender	4	Was the researcher male or female?	Methods – Data Analysis (research team)
Experience and training	5	What experience or training did the researcher have?	Methods – Data Collection (training by FM and SM; university courses on qualitative methods and interviewing)
Relationship established	6	Was a relationship established prior to study commencement?	No; Methods – Data Collection
Participant knowledge of the interviewer	7	What did the participants know about the researcher?	Methods – Data Collection (dual role not disclosed during interviews)
Interviewer characteristics	8	What characteristics were reported about the interviewer/facilitator?	Methods – Data Collection; Limitations (physiotherapy background)
**Domain 2: Study design**			
Methodological orientation and theory	9	What methodological orientation was stated to underpin the study?	Methods – Data Analysis (structuring qualitative content analysis, Kuckartz)
Sampling	10	How were participants selected?	Methods – Study Design (consecutive sampling of eligible patients)
Method of approach	11	How were participants approached?	Methods – Study Design (face-to-face in consultations; interview invitation by e-mail)
Sample size	12	How many participants were in the study?	Results (n = 10; 16 interviews)
Non-participation	13	How many people refused to participate or dropped out? Reasons?	Results – participant flow; Supplementary dropout questionnaire (screening numbers not systematically recorded)
Setting of data collection	14	Where was the data collected?	Methods – Data Collection (telephone; participants in a private setting of their choice)
Presence of non-participants	15	Was anyone else present besides the participants and researchers?	No; Methods – Data Collection
Description of sample	16	What are the important characteristics of the sample?	Results, Tables 1–2
Interview guide	17	Were questions, prompts, guides provided by the authors? Was it pilot tested?	Methods – Data Collection; Supplementary material (pilot tested in two interviews)
Repeat interviews	18	Were repeat interviews carried out? If yes, how many?	Yes; six follow-up interviews (Methods – Study Design)

Audio/visual recording	19	Did the research use audio or visual recording to collect the data?	Methods – Data Collection (audio)
Field notes	20	Were field notes made during and/or after the interview or focus group?	Not taken
Duration	21	What was the duration of the interviews or focus group?	Results (mean 13 min 35 s; range 7 min 34 s – 19 min 46 s)
Data saturation	22	Was data saturation discussed?	Saturation not used as a stopping criterion; sample-size rationale based on information power (Methods – Study Design)
Transcripts returned	23	Were transcripts returned to participants for comment and/or correction?	Not performed
**Domain 3: Analysis and findings**			
Number of data coders	24	How many data coders coded the data?	One (SB); Methods – Data Analysis
Description of the coding tree	25	Did authors provide a description of the coding tree?	Supplementary material (coding tree and coding guideline)
Derivation of themes	26	Were themes identified in advance or derived from the data?	Methods – Data Analysis (main categories deductive; subcategories inductive)
Software	27	What software, if applicable, was used to manage the data?	MAXQDA 24; Methods – Data Analysis
Participant checking	28	Did participants provide feedback on the findings?	Not performed
Quotations presented	29	Were participant quotations presented to illustrate the themes/findings? Was each quotation identified?	Results (quotations identified by participant ID and interview time point, T0/T1)
Data and findings consistent	30	Was there consistency between the data presented and the findings?	Results
Clarity of major themes	31	Were major themes clearly presented in the findings?	Results
Clarity of minor themes	32	Is there a description of diverse cases or discussion of minor themes?	Results (negative cases; non-completers; Table 3)

Developed from: Tong A, Sainsbury P, Craig J. Consolidated criteria for reporting qualitative research (COREQ): a 32-item checklist for interviews and focus groups. Int J Qual Health Care 2007;19(6):349–357.
