# Supplementary material for: Patient experiences with a prescription-based digital health application (DiGA) for knee disorders: a qualitative study in german orthopaedic care
Source: BMC Musculoskelet Disord. 2026 Oct 28;27:953. doi: 10.1186/s12891-026-10550-4 (PMC13733015; doi:10.1186/s12891-026-10550-4)
Supplement: Supplementary file 2 [file 12891_2026_10550_MOESM1_ESM.docx]

**Interview Guide – DiGA@Ortho: First Impressions**

| Key questions/ Story prompt | Checkpoints | | Specific questions | | Maintenance and control issues |
| --- | --- | --- | --- | --- | --- |
| Your doctor has prescribed you a DiGA (digital health application), in other words, an ‘app on prescription’.  I would like to ask you a few questions about the prescription process. | App  Indications  Prescription process  Information | | (What is the app called?)  What are your expectations of the app? What kind of content would you like to see?  How did the prescription process go?  Who prescribed the app for you?  What did the doctor tell you?  How could the prescription process be improved? | | Could you tell us a bit more about that?  And then?  What was that like for you?  What’s your view on that? Could you please elaborate?  Could you give an example of that, please?  What do you mean by that, specifically?  Could you tell us a bit more about that?  And then?  What was that like for you?  What’s your view on that? Could you please elaborate?  Could you give an example of that, please?  What do you mean by that, specifically? |
| Now I would like to talk to you about your expectations regarding the DiGA. | Expectations/Challenges/Hopes  General attitudes towards digitalisation in the healthcare system  Issues relating to alternative care provision | | What do you expect from the app?  What do you hope to achieve by using the app?  What challenges do you think you might face when using the app?  Do you use any other health apps (running app, fitness app, meditation app, sleep app, etc.)?  What is your opinion on the digitalisation of the healthcare system?  Have you consented to your data being stored in the electronic patient record (ePA)?  Could you imagine that using an app might complement physiotherapy? If not, why not?  Could you imagine that using an app might replace physiotherapy? If not, why not? | |  |
| **Age** | | **Gender** | | **App name** | |
|  | |  | |  | |
| **underlying condition** | | **Occupation** | | **Highest level of education** | |
