# Supplementary material for: Patient experiences with a prescription-based digital health application (DiGA) for knee disorders: a qualitative study in german orthopaedic care
Source: BMC Musculoskelet Disord. 2026 Oct 28;27:953. doi: 10.1186/s12891-026-10550-4 (PMC13733015; doi:10.1186/s12891-026-10550-4)
Supplement: Supplementary file 3 [file 12891_2026_10550_MOESM2_ESM.docx]

**Interview Guide – DiGA@Ortho**

| Key question / Story prompt | Checkpoints | Specific questions | Maintenance and control issues |
| --- | --- | --- | --- |
| You have been using a digital health app – that is, an app for your healthcare – for some time now.  Could you please describe the app? | App  Indications  Prescription process  Information  Installation | How did the registration go? Did everything go smoothly?  How long did it take to receive the code from your health insurance provider?  If physiotherapy was prescribed, how long did it take before you had your first physiotherapy session?   - How did you find it booking appointments for physiotherapy? | Could you tell us a bit more about that?  And then?  What was that like for you?  What’s your view on that? Could you please elaborate?  Could you give an example of that, please?  What do you mean by that, specifically? |
| Now I’d like to talk to you about how to use the app | User experience  Frequency  Preferences  Usability  Handling  Functionality  Room for improvement  Sustainability  Incentives | How often have you used the app so far?  Have you used the app at a consistent rate? Or sometimes more, sometimes less?  How did you find using the app?  What worked well? Please describe.  What didn’t work well? What bothered you?  How did the app help you achieve your goal?  What advantages do you see in apps as an additional treatment option?  What would you change or improve about the app? What is missing?  Would you like to continue using the app?  Have you used all the features in the app?  Would you use the app more often? Why / why not?  What would motivate you to use the app more often?  Were there any uncertainties regarding the exercises?  What feedback does the app provide after an exercise?  Is there anything else you’d like to add? |  |
