# Supplementary material for: Patient experiences with a prescription-based digital health application (DiGA) for knee disorders: a qualitative study in german orthopaedic care
Source: BMC Musculoskelet Disord. 2026 Oct 28;27:953. doi: 10.1186/s12891-026-10550-4 (PMC13733015; doi:10.1186/s12891-026-10550-4)
Supplement: Supplementary file 4 [file 12891_2026_10550_MOESM3_ESM.docx]

**How to: Prescribing Digital Health Applications (DiGA)**

If a DiGA is listed in the register of the Federal Institute for Drugs and Medical Devices (BfArM), it is covered by health insurance funds. For knee joint injuries, the following three DiGAs are currently available:

**-	companion patella powered by medi – approved by the German Knee Society**

**-	Mawendo**

**-	Orthopy for knee injuries**

Below are the ICD-10 diagnoses published by the manufacturers and the BfArM that could lead to a prescription, along with the *age groups* and contraindications:

|  | **companion patella powered by medi - proved by Dt. Kniegesellschaft** | **Mawendo** | **Orthopy for knee injuries** |
| --- | --- | --- | --- |
| **Indications**  *Age groups* | **M22.2** Diseases of the patellofemoral region  **M22.4** Chondromalacia patellae  **M76.5** Tendinitis of the patellar tendon  **M79.66** Pain in the extremities: lower leg [fibula, tibia, knee joint]  **S83.0** Dislocation of the patella  *Adolescent(s) (14–17 years)*  *Adult(s) (18–65 years)* | **M22** Diseases of the patella  *Adolescents (aged 12–17)*  *Adults (aged 18–65)*  *Adults (over 65)* | **M23.2** Meniscal damage due to an old tear or old injury  **M23.3** Other meniscal damage  **M23.61** Other spontaneous rupture of one or more ligaments of the knee joint: anterior cruciate ligament  **S83.2** Meniscus tear, acute  **S83.53** Sprain and strain of the knee joint: tear of the anterior cruciate ligament  *Adult(s) (18–65 years)* |
| **Contraindications** | **M00** Purulent arthritis  **M01** Direct joint infections associated with infectious and parasitic diseases classified elsewhere  **M22.0** Habitual dislocation of the patella  **M23.4** Loose body in the knee joint  **M93.26** Osteochondrosis dissecans: lower leg [fibula, tibia, knee joint]  **S83.1** Dislocation of the knee joint  **S83.2** Acute meniscal tear  **S83.3** Acute tear of the knee joint cartilage  **S83.4** Sprain and strain of the knee joint involving the (fibular) (tibial) collateral ligament  **S83.5** Sprain and strain of the knee joint involving the (anterior) (posterior) cruciate ligament  **S83.7** Injury to multiple structures of the knee | **A40** Streptococcal sepsis  **A41** Other sepsis  **A48** Other bacterial diseases, not elsewhere classified  **F23** Acute transient psychotic disorders  **I00** Rheumatic fever without specified cardiac involvement  **I01** Rheumatic fever with cardiac involvement  **I20** Angina pectoris  **I21** Acute myocardial infarction  **I24** Other acute ischaemic heart disease  **I26** Pulmonary embolism  **I50** Heart failure  **I51** Complications of heart disease and unspecified heart disease  **J12** Viral pneumonia, not elsewhere classified  **J13** Pneumonia caused by Streptococcus pneumoniae  **J14** Pneumonia caused by Haemophilus influenzae  **J15** Bacterial pneumonia, not elsewhere classified  **J16** Pneumonia caused by other infectious agents, not elsewhere classified  **J17** Pneumonia associated with diseases classified elsewhere  **J18** Pneumonia, unspecified causative agent  **M00** Septic arthritis | **I26** Pulmonary embolism  **I50** Heart failure  **I80.2** Thrombosis, phlebitis and thrombophlebitis of other deep vessels of the lower extremities  **J18** Pneumonia, unspecified aetiology  **M00** Septic arthritis  **M01** Direct joint infections associated with infectious and parasitic diseases classified elsewhere  **M22.0** Habitual dislocation of the patella  **M23.4** Loose body in the knee joint  **M24.07** Loose body: ankle and foot [tarsus, metatarsus, toes, ankle joint, other joints of the foot] |

Here’s a quick overview of what the DiGAs offer:

| **companion patella powered by medi - proved by Dt. Kniegesellschaft** | **Mawendo** | **Orthopy for knee injuries** |
| --- | --- | --- |
| - A movement therapy training plan (tailored to your pain levels and physical capacity) - A specialist library containing educational articles on your condition - A visual representation of pain and functional limitations | - Exercise programme with instructional videos **(the programme is initially selected by a doctor!)** - Health information - Documentation options | - Physiotherapy training plans featuring exercises in line with clinical guidelines (intensity is assessed based on severity, heat build-up at the affected site and pain perception) - Easy-to-understand educational articles - Visualisation of progress made during therapy to help motivate patients |

**Regulatory pathway**

2 ways to obtain a prescription: - Prescription issued following a decision by a doctor or psychotherapist - Application to the health insurance fund

Prescriptions on Form 16 (used, among other things, for medical aids) --> from 1 January 2025, these will only be issued electronically, as with e-prescriptions

Special features:- The prescription duration does not need to be specified*- Unique pharmaceutical identification number (PZN)**

Insured persons should then contact their health insurance provider, which will generate a prescription code (a string of characters plus a QR code); they can then use this code to download the app from the App Store. The costs for the digital health application (DiGA) will then be settled directly between the health insurance provider and the manufacturer! There is no co-payment required for insured persons.

* Companion Patella: 90 days // Mawendo: 360 days (single-use licence) // Orthopy: 365 days (single-use licence)

** PZN: Companion Patella 17850263 // Mawendo 17622734 // Orthopy 1907639
