# Supplementary material for: Patient experiences with a prescription-based digital health application (DiGA) for knee disorders: a qualitative study in german orthopaedic care
Source: BMC Musculoskelet Disord. 2026 Oct 28;27:953. doi: 10.1186/s12891-026-10550-4 (PMC13733015; doi:10.1186/s12891-026-10550-4)
Supplement: Supplementary file 5 [file 12891_2026_10550_MOESM4_ESM.docx]

**Supplementary Table: Responses of participants who withdrew from the study**

**(N = 4)**

| Item | Response option | n(%) |
| --- | --- | --- |
| **Q1: Was the information about the DiGA study (e.g. the study’s objectives, procedure, data protection) clear and easy to understand?** | Yes, everything was clear and easy to understand  Some of the information was unclear  No, a lot of the information was unclear or incomplete  No response | 3 (75%)  1 (25%)  0 (0%)  0 (0%) |
| **Q2: Why did you stop using the DiGA? (You may select more than one option)** | The app was technically difficult to use  The DiGA’s features did not meet my expectations  I didn’t have time to use the DiGA regularly  The DiGA did not provide sufficient support for my health needs  I did not feel secure or comfortable with the health data  The DiGA caused technical problems or errors  I did not notice any positive changes or improvements from using it  I did not find the DiGA motivating enough  Other reasons (please specify): | 0 (0%)  0 (0%)  2 (50%)  1(25%)  0 (0%)  0 (0%)  0 (0%)  1 (25%)  0 (0%) |
| **Q3: Did you encounter any technical difficulties when using the DiGA?** | Yes, lots of problems  Yes, a few problems  No, no problems  No answer | 0 (0%)  0 (0%)  3 (75%)  1 (25%) |
| **Q4: Have you received support through the DiGA in relation to your health needs?** | Yes, very helpful  Yes, somewhat helpful  No, not helpful  I haven’t noticed any change  No answer | 1 (25%)  1 (25%)  2 (50%) |
| **Q5: What was the reason for not participating in/continuing the study?** | Health-related reasons (e.g., illness, worsening symptoms)  Lack of time and resources  Not sufficiently informed about the potential benefits of the study  Adequate physiotherapy already available  No further details provided | 0 (0%)  1 (25%)  1 (25%)  1 (25%)  1 (25%) |
