# Supplementary material for: Patient experiences with a prescription-based digital health application (DiGA) for knee disorders: a qualitative study in german orthopaedic care
Source: BMC Musculoskelet Disord. 2026 Oct 28;27:953. doi: 10.1186/s12891-026-10550-4 (PMC13733015; doi:10.1186/s12891-026-10550-4)
Supplement: Supplementary file 6 [file 12891_2026_10550_MOESM6_ESM.docx]

| Category name | Category name | Category name | Category name |
| --- | --- | --- | --- |
| **Acceptance** | Acceptance and integration with regard to digital health applications (DiGA) and digitalisation, but also willingness to undergo therapy; more specifically:   - How positively is the innovation perceived? - What attitudes, experiences or personal factors influence this willingness? | *“Prescribing the app was a new experience for me. I’d never had a prescription from the hospital before that specifically stated it should be sent to the health insurance company. So that’s what I did, and I just waited to see what would happen. Normally, I take a prescription to the chemist.”*  *(paraphrased, ID7, pos. 12–13)* |  |
| **Potential** | Opportunities to use existing resources to achieve a specific goal; more specifically:   - What opportunities do patients see in the use of digital health apps? - Have goals been achieved through the use of DiGA? - Where have gaps in care been closed? - How do patients experience the use of a DiGA as a complementary or alternative measure to standard care (usually physiotherapy)? | *“I had a positive feeling about it. I think it’s good that this option is available and that I’ve had the chance to give it a go. (.) It’s good for other people too, because it can be difficult to find a physiotherapist for outpatient treatment. If you have the option of doing the exercises at home, that’s really good.”*  *(paraphrased, ID2, pos. 13)* |  |
| **Challenges** | A task requires a certain amount of effort, knowledge and perseverance to overcome. Any obstacles, risks, negative experiences or structural problems that stand in the way of the successful use of a digital health application (DiGA); more specifically:   - What was difficult about using the DiGA? | *“But having that kind of oversight from another person – seeing that ‘it’s not quite working properly yet, let’s try doing it this way’ – I don’t think any machine or programme can replace that at the moment, at least.”*  *(ID1, pos. 38)*  *“When it comes to using the app, I could imagine it being challenging to get an overview at first. How does the app work? Where do I find things? (..) It’s uncharted territory at first. (.) You have to click your way through it a bit to see how it works. Where do I find what?”*  *(paraphrased from ID2, pos. 22–23)* | *Following analysis of the interviews in this category, this also includes code activation by the health insurance provider*  *This may also include negative aspects relating to acceptance and potential. The distinction is made on the basis of the negative connotation.* |

| Subcategory: **Acceptance** | Definition | *Key quote* | Coding rule |
| --- | --- | --- | --- |
| **Installation and user-friendliness/usability** | All comments relating to the initial set-up of the DiGA and the day-to-day use of the application.  More specifically:   - Aspects of the user interface (e.g. menu structure, navigation, clarity) - Technical issues (e.g. long loading times, compatibility) - Perceived ease of use or complexity. | *“The exercise selections are pretty cool in themselves. And the fact that there’s always a video to go with them, so you know what the exercise is and so on. I think that’s particularly valuable for people who are new to this topic. Apart from that, the app itself is cool and easy to use.”*  *(paraphrased from ID1, pos. 29)* | *Following analysis, interaction with the health insurance provider has been deliberately classified as a challenge*  *Distinction from:*   - *Content and therapeutic exercises → fall under DiGA content/training development* - *Data protection or security concerns → fall under electronic patient records, data security* - *Motivation through the app → falls under potential/motivation* |
| **Frequency and duration of use** | All statements relating to how often (e.g. daily, weekly, irregularly) and for how long (e.g. over weeks, months, discontinued after a short time) the digital health application was actually used  Specifically:   - Duration, continuity and intensity of use | *“S1: And how long have you been using the app in total now?*  *S2: I’d say three to four weeks.*  *S1: And have you used the app at the same frequency all along, or did it depend on anything?*  *S2: Well, apart from one day, I’ve actually used it every day.”*  *(ID9, lines 48–51)* | *This isn’t about the reasons*   - *positive (motivation) → potential/motivation* - *negative (frustration, technical problems) → challenges/negatives and criticism* |
| **Use of other health apps** | All statements in which participants report using other digital health applications (apart from the DiGA under investigation). These include fitness apps, step counters, meditation apps, blood glucose diaries, other rehabilitation or training apps, etc. The focus is on experience with and attitudes towards other apps as an indicator of general willingness to use digital tools in a healthcare context. | *“A pedometer, like probably everyone else. Then there’s a health app from my health insurance provider, which also offers digital services. Other than that, I don’t really use any other apps. No weight-loss apps or fasting apps or anything like that, which other people still use.”*  *(paraphrased, ID3, pos. 19)* |  |
| **an affinity for technology** | Any statements relating to general attitudes, skills or experience in using digital technologies (e.g. smartphones, computers, apps) – regardless of whether they are directly related to the DiGA.  Specifically:   - a positive openness towards and enjoyment of technology - scepticism, uncertainty or feeling overwhelmed when using digital media. | *“Well, to be perfectly honest, I’m generally very open-minded when it comes to technology. So I’m unbiased and tend to have a positive attitude towards anything that means I don’t have to go out of my way to get things.”*  *(ID1, pos. 46)* | *Analytic memo (not a coding rule): possible associations, for example between technical proficiency and acceptance, are examined during interpretation and must not be assumed during coding.* |
| **Views on digitalisation in the healthcare system** | All statements relating to the general assessment and evaluation of digitalisation in the healthcare sector – regardless of the specific use of digital health applications (DiGA).  More specifically:   - Views on topics such as modernisation, opportunities, risks, scepticism towards digital structures, comparisons with the existing system, or political and regulatory frameworks. | *“S1: What is your view on digitalisation in the healthcare system?*  *S2: I think it’s important for speeding up processes. It’s also a more efficient way of clarifying matters more quickly than through traditional methods, such as postal correspondence or the like. So I’m very open to it. (.) And I also believe that this is the right way forward.”*  *(ID3, lines 20–21)* |  |

| Subcategory: Potential | Definition | *Key quote* | Coding rule |
| --- | --- | --- | --- |
| **Physiotherapy and DiGA interaction**   - **Complementing or replacing physiotherapy** | All statements describing how the DiGA is perceived or used in the context of physiotherapy care.   - All statements in which the DiGA is used in addition to physiotherapy treatment to support exercises, motivation or the course of therapy, without replacing traditional therapy. - All statements in which the DiGA is used in place of physiotherapy treatment, either as a substitute for appointments or to reduce the number of physical therapy sessions. | *“But of course, on days when I can’t go out, I can actually get my knee moving using the app. So I don’t have to miss out on any exercise. Whether these end up being the same exercises as those in physiotherapy is something we can incorporate later on as well.*  *“I’d see that as a good combination – linking the app with physiotherapy in equal measure. Combining the exercises.”*  *(Paraphrased from ID6, pos. 46)* | *Currently still divided into the two areas of ‘supplementing’ and ‘replacing’ physiotherapy; in Germany, DiGA is planned as a supplement, hence the somewhat provocative question and the suggestion that participants should distinguish more clearly between DiGA and physiotherapy* |
| **Expectations regarding the DiGA** | All statements in which participants describe the effects, functions or improvements they hope to achieve by using the digital health application. These refer to expectations held before or during use – regardless of whether those expectations were met. | *“Little bits of help like that, or a better understanding of what’s been done and how best to get back on my feet. In the truest sense of the word.” (ID3, 15)*  *“I always have very low expectations. I just let things take their course. I’d be happy if someone could guide me a bit and show me options, give me exercises, so that perhaps something about my problem might improve.” (ID2, pos. 15)* |  |
| **General benefits of a DiGA** | All statements describing the positive effects, benefits or advantages of the DiGA.  Specifically:   - captures the general perceived potential of the digital application | *“It’s not just useful for recovery, but also for long-term improvement and strengthening the knee. (.) It’s clear that you won’t return to your original condition in one, two or three weeks, but that it’s a longer process. That’s why I’d continue to use the app. Sure, you can also watch exercises on YouTube, but this is much more concise and explained in a more professional way.”*  *(ID3, pos. 38–41)* |  |
| **Motivation and goals associated with the use of digital health apps** | Any statements describing why patients use the digital health application and what specific goal or outcome they are aiming for. To be more specific in terms of adherence:   - To what extent has the digital health application helped you to stick to the exercises? | *“For me, this is something I can hold on to. I always need someone like that to guide me a little. Someone who tells me to do these particular exercises, as they’re good for my body. And that’s what helps me. Because that way, I stick with it. And the exercises I’m doing now cause me no pain, or very rarely.”*  *(Paraphrased from A17, 32–33)* | *Later on, thematic links between motivation, goal-setting and actual usage can be identified.* |
| **DiGA content / Training development** | All statements relating to the content, training structure and type of exercises in the DiGA. Specifically:   - Selection of training programmes - Adaptation to individual needs - Educational presentation (e.g. videos, instructions, feedback mechanisms) - What criteria are used to select the exercises? | *“I put the training programme together by looking at the exercises available and deciding which ones were suitable for me. I chose the exercises myself. I have some background knowledge of the fitness industry and discussed it with my physiotherapist.”*  *(paraphrased, ID1, pos. 14–17)* | *crucial for later discussing the potential for personalised treatment approaches.* |

| Subcategory: Challenges | Definition | *Key quote* | Coding rule |
| --- | --- | --- | --- |
| **Medical consultation and the prescription process** | All statements relating to medical advice, information about digital health applications (DiGA) and the prescription process. | *“They didn’t give me any information at all about what it was actually about, though. I would have liked them to have briefly explained what it was. I didn’t get the impression that they were particularly well-informed. Otherwise, it was actually self-explanatory.”*  *(ID5, pos. 13)* | *Due to the high volume of cases, dealing with the health insurance provider falls into the category of administrative delays* |
| **Electronic health records / Data security** | All statements regarding data protection, data security and electronic documentation in connection with the DiGA.  Specifically:   - Trust in digital data management, concerns regarding data security, interfaces with the electronic patient record. | *So yes, lots of people always say things like, ‘Yeah, but the data and so on,’ but what are they supposed to do? Ultimately, the fact is that I haven’t signed anything or anything like that. So in that sense, yes, they’re allowed to use my data, but I still find it hard to imagine that anything negative would actually happen – I haven’t had any negative experiences yet – that my data would be used for anything harmful.*  *(paraphrased, ID2, pos. 45)* |  |
| **Administrative delay** | Any information regarding delays, whether due to timing or organisational issues, that affect access to the DiGA. In particular, the time taken for the activation code to be made available by the health insurance provider. | *“S1: How long did it take from the prescription being issued to receiving the health insurance code? S2: I think it was about ten working days, so two weeks, roughly ten working days.”*  *(ID5, lines 8–9)* |  |
| **Practice and patient safety** | Any statements relating to risks or uncertainties associated with performing the exercises.  Specifically:   - Risks arising from incorrect execution of the exercises, failure to adapt them sufficiently to individual abilities, and health risks. | *“The app also explains that you may experience some cracking and crunching, as well as mild discomfort, at first. No one goes beyond their normal limits. As for what is bearable or beneficial for oneself, anyone in the best of health knows that you don’t slide around on your knees if you’ve had knee surgery. I’m of the opinion that I think the app is good. But you just have to take the time.”*  *(ID3, Pos. 34-35)* |  |
| **Negative aspects and criticism regarding the specific app** | Any comments relating to general negative experiences, dissatisfaction or criticism of the app itself.  Specifically:   - Technical issues - Inadequate content - Poor user guidance - Lack of usefulness | *“If I give feedback, I’d like to see it lead to something. So if I were to say that the exercises are too easy for me, I’d personally think it would be really cool if a few alternative exercises were provided as well. In other words, if this feedback loop were to result in me being given new exercises as well.”*  *(paraphrased, ID5, pos. 46)* |  |
